# Chinese caregivers’ preferences and predicted uptake of HPV vaccination: a study protocol for two discrete choice experiments

**DOI:** 10.1136/bmjopen-2024-089565

**Published:** 2026-01-08

**Authors:** Han Fang, Jialing Mu, Eric PF Chow, Jason J Ong, Dan Wu, Ye Zhang

**Affiliations:** 1 National Vaccine Innovation Platform & State Key Laboratory of Reproductive Medicine and Offspring Health & Department of Social Medicine and Implementation Science, School of Public Health, Nanjing Medical University, Nanjing, Jiangsu, China; 2 Melbourne Sexual Health Centre, Carlton, Victoria, Australia; 3 Monash University Faculty of Medicine Nursing and Health Sciences, Melbourne, Victoria, Australia; 4 London School of Hygiene & Tropical Medicine, London, UK; 5 University of New South Wales, Sydney, New South Wales, Australia

**Keywords:** HPV Vaccine, Caregivers, China, Public health, SOCIAL MEDICINE

## Abstract

**Abstract:**

**Introduction:**

Human papillomavirus (HPV) vaccines have been introduced in the Chinese market since 2016. However, the HPV vaccine coverage is still remarkably low among adolescent girls in China. This study will employ discrete choice experiments (DCEs) to elicit the preferences of Chinese caregivers for HPV vaccine-related profile characteristics and service delivery methods for adolescent girls.

**Methods:**

Two DCEs will be conducted with caregivers of girls aged 9–18 in China. The first DCE will focus on caregivers’ preferences regarding the HPV vaccine-related profile for girls aged 9–18, with potential attributes including level of protection against cervical cancer, level of protection against genital warts, risk of severe side effects, number of dose(s), place of manufacture, waiting time and cost for one dose. The second DCE will assess Chinese caregivers’ preferences for vaccination service delivery methods, with the potential attributes including source of recommendation, information channel, message framing, how to make an appointment, location for receiving the vaccine and incentives. A total of 300 participants will be recruited to complete the DCEs. We will summarise the key socio-demographic characteristics of participants and use latent class and mixed logit models to assess preferences and preference heterogeneity in HPV vaccination services.

**Ethical and dissemination:**

Ethical approval was obtained from the Research and Ethics Committee at Nanjing Medical University. Findings from this study will be disseminated widely to relevant stakeholders via scheduled meetings, webinars, presentations at conferences and in peer-reviewed journal manuscripts.

**STRENGTHS AND LIMITATIONS OF THIS STUDY**

- This study employs a discrete choice experiment (DCE) to inform the design of a kindness-based human papillomavirus (HPV) vaccination intervention.
- This study applies the DCE to identify modifiable service attributes that could substantially increase HPV vaccine uptake to levels consistent with guideline recommendations.
- The study will be conducted in two provinces in China with relatively higher economic levels. Participants will be recruited using convenience sampling, which may limit the generalisability of the findings to other settings or populations.

## Introduction

 Cervical cancer caused by infection with human papillomavirus (HPV) poses a considerable threat to women’s health. Although most cases are preventable with HPV vaccination,^1^ a global estimate indicates 604 000 new cases and 342 000 cervical cancer-related deaths in 2020.^2^ The WHO launched an initiative to expedite the elimination of cervical cancer.^3^ China accounts for approximately one-fifth of new cervical cancer diagnoses worldwide every year.^2^ In addition, the age-standardised incidence rate of cervical cancer in China has been showing an upward trend, with an annual increase of 1.5% between 1988 and 2017.^2 4^ This underscores the urgency to improve the HPV vaccination coverage rate in China.

The HPV vaccine was first introduced in China in 2016 and approved for use among women aged 9 to 45 years by the China Food and Drug Administration. Currently, there are five different vaccines available in China, namely Cecolin (domestic HPV-2), Cervarix (imported HPV-2), Gardasil (imported HPV-4), Gardasil9 (imported HPV-9) and a new domestically produced bivalent vaccine (Walrinvax). However, after HPV vaccines became available in China for 7 years, the vaccination coverage rate remains extremely low; the vaccine coverage rate among WHO’s recommended primary target population (girls aged 9–14 years old) is less than 1%, far behind the WHO recommended target of 90%.^5^ Previous studies found that parents’ low HPV-related literacy, low perceived risk of cervical cancer and concerns about vaccine safety and efficacy may influence their willingness to have their children vaccinated.^6 7^ Besides these individual-level factors, China also faced several structural-level challenges in implementing HPV vaccination for eligible girls. First, high prices for HPV vaccines remain a significant obstacle. HPV vaccines are not included in China’s national immunisation programme and associated fees are prohibitive for many.^8^ In addition, there is insufficient supply of imported HPV vaccines in China, and the waiting time for imported 4-valent and 9-valent vaccines often exceeds 6 months.^9^ While the WHO has recommended a one-dose HPV vaccine regimen for women aged 9 to 20, the Chinese guideline still adopts a two to three-dose schedule.

Chinese scientists have suggested that adapting to a reduced schedule of HPV vaccination could help address both supply-related issues and reduce vaccination costs in China.^10^ However, limited evidence exists regarding the role of vaccine availability and dose schedule on caregiver decision-making process when it comes to having their children vaccinated. To improve the coverage of HPV vaccination in China, the Chinese government encouraged the development of innovative programmes. Fifteen provinces and cities have initiated pilot programmes since early 2021, and most adopted financial incentive strategies, offering either free or fixed financial subsidies to girls aged 13 to 14 years to receive up to two doses.^11^ Innovative strategies are also being piloted, for example, ‘pay-it-forward’ in which girls received subsidised vaccines and were given an opportunity to donate to help other girls to get vaccinated, showed preliminary effectiveness.^12^ Furthermore, the HPV vaccine has been delivered in various settings among these pilot programmes, such as schools or local community health service centres. To optimise HPV vaccine service delivery and resource allocation, it is important to understand caregivers’ preferences for HPV vaccine services.

Cervical cancer ranked second among female cancer incidences in Jiangsu Province and sixth in Zhejiang Province.^13 14^ To reduce this disease burden and advance HPV vaccination efforts, both Jiangsu and Zhejiang began offering free domestic bivalent HPV vaccinations to eligible 14-year-old girls in 2022, which substantially increased the HPV vaccination rate among this age group. However, the current vaccination efforts face two prominent challenges. First, the vaccination rate among first-grade junior high school girls is gradually reaching a plateau, creating a pressing need to surpass the current coverage level. Second, the existing policy exclusively targets 14-year-old adolescent females, leaving a gap in mobilisation mechanisms for girls aged 15 years, whose participation also requires focused attention. These practical challenges highlight the urgency and necessity of conducting in-depth research in pioneering cities like Nanjing and Hangzhou to optimise service delivery models and resource allocation efficiency.

Previous studies examined parents’ preferences for HPV vaccination,^6 15^ but these are old and did not include real-world evidence, availability and supply, and recent international guidelines on dosage in their attribute design. This study will first examine caregiver preferences for HPV vaccine-related profile characteristics. Second, we will also explore caregiver preferences for vaccine service delivery models which may have implications for improving vaccine programme implementation and enhancing efficiency.

## Methods and analysis

### Overview of approach and methods

We will use two discrete choice experiments (DCEs) to investigate Chinese caregiver preferences regarding HPV vaccination and its related service delivery for their daughters aged between 9–18 years old. DCE elicits stated preferences, with substantial application in healthcare to facilitate the design of user-centred health services.^16^ Based on random utility theory, DCE assumes that an individual’s choice is rational and will always select the option that provides them with the highest utility.^16^ In addition, DCE is also based on Lancaster’s theory, which suggests that the utility of a good or service can be defined by different characteristics or attributes of that good or service.^16^ In a DCE, each participant faces a series of choice sets (in a choice task, the complete set of options presented to respondents for trade-off comparisons, usually with two alternatives). Each choice set consists of several attributes (ie, the characteristics of an option, such as ‘cost’) that ask respondents to trade off between preferred and less preferred attribute levels (ie, the specific values that each attribute takes, such as ‘CNY300’, ‘CNY800’) present in each choice task. Therefore, the choice data generated can elicit the relative importance of attributes in decision-making, the trade-offs the respondents are willing to make and the impact on uptake if attribute levels are changed.

### Consumers and public involvement

Consumers and key stakeholders will be involved in finalising the attributes for the DCE. Their input will provide valuable feedback on the relevance, clarity and classification of potential attributes, ensuring that these attributes accurately reflect their needs and perceptions. They will also contribute to optimising the final presentation of the questionnaire, making it clearer and easier to understand.

### Phase I—semi-structured interviews

Qualitative data will be gathered through a total of four semi-structured group discussions, with 6–8 interviewees for each group discussion (the interview outline can be found in the online supplemental file 1). Study participants will be purposively sampled among caregivers of eligible girls (9–18 years) who have either been vaccinated or plan to receive the HPV vaccine. Social media platforms and health information systems of community health centres will be used to recruit participants. Individuals expressing interest will be contacted by the research team skilled in community engagement and qualitative research. Interviews will be conducted through either online or in-person meetings, depending on participant preference. The online group will use professional video conferencing software (Tencent Meeting) to conduct the session. The host will control the flow, and the chatroom and raise hand features will simulate in-person interaction. The primary focus of the group discussion is to collect information on the factors influencing participants’ decision-making processes and the relevant importance of these factors regarding HPV vaccine services.

All interviewees will be asked to provide online informed consent. The group discussions will be recorded after obtaining participant consent. Prior to the discussion, verbal consent will be reconfirmed from each participant. The group discussions will last 60–90 min and will be conducted in Mandarin. Each participant will receive an honourarium worth US$21 in appreciation for their participation.

### Qualitative data analysis

The group discussion will be audio-recorded, and we will transcribe the audio file verbatim in Mandarin. We will employ thematic analysis to analyse qualitative data, using both inductive and deductive methods for coding. We will develop a coding framework using the first two transcripts. Potential themes will be explored based on previous literature, and emerging new themes from raw data will also be generated. The potential themes include vaccine cognition (eg, respondents’ prior understanding of HPV vaccines), price and subsidy (eg, whether vaccine prices are a barrier to vaccination, acceptable price ranges, attitudes toward economic subsidies and preferred subsidy forms) and community engagement and support for other people. Two coders will then review all transcripts and apply the coding framework to analyse the data independently, using NVivo to organise the data. New codes and themes will be allowed. The two coders will thoroughly examine coding schemes and resolve discrepancies in data coding. In cases of disagreement, a third coder with substantial qualitative experience will be invited to discuss code definitions and applicability until a consensus is reached. Themes can be understood as summaries highlighting the most prominent issues raised by participants on a specific topic. Data saturation is defined as identifying points of repeated themes among participants in the absence of new discoveries. Themes, along with informative quotations from participants, will be translated to English.

### Data management

Each respondent will be assigned a study number. All data collected during the course of the research will be kept confidential and accessible only to the coders and data manager. All informed consent forms and survey data will be digitised, while non-digital data formats will be stored in secure cabinets at the local institution (Public Health School of Nanjing Medical University).

### Phase II—discrete choice experiment survey

#### Developing the DCE design

##### Experimental design

**Figure 1:**
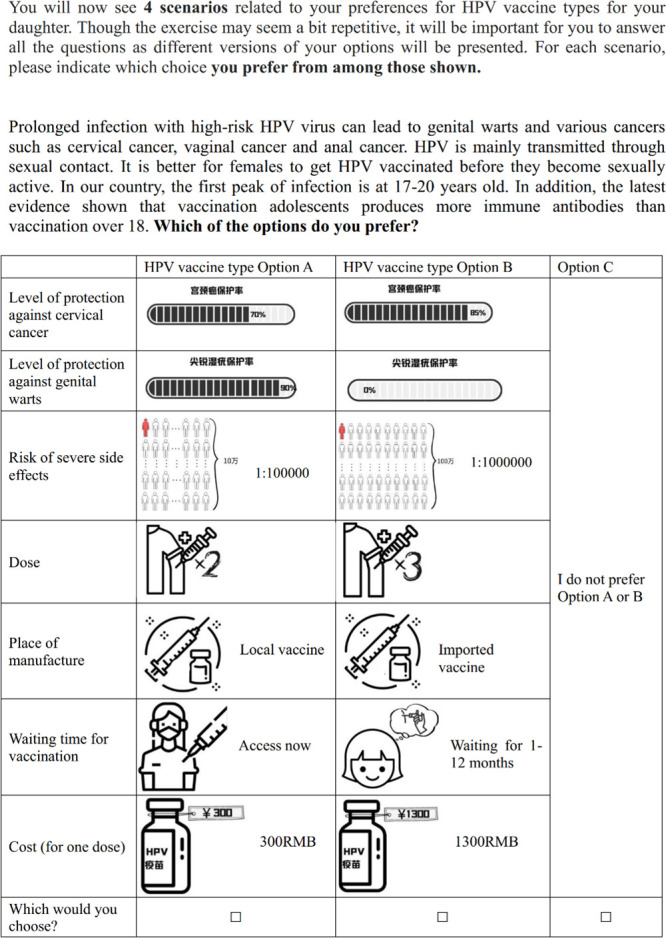
Example of a choice scenario in the discrete choice experiment (A). This scenario presents a hypothetical choice set from the HPV vaccine discrete choice experiment, illustrating the attributes and levels presented to study participants for evaluation. HPV, human papillomavirus.

Our team has conducted a comprehensive review of (1) the global-focused and China-focused published literature to inform the preference for HPV vaccination and its related services (unpublished data); and (2) the potential strategies and approaches of HPV vaccination (the search parameters and findings can be found in online supplemental file 1). With these, we have compiled a draft list of attributes and their associated levels (tables1  2) but these will be optimised based on our subsequent group discussions and expert consultations. We will engage experts from multiple fields (eg, the DCE design experts and sexual health experts), policymakers from the local health departments and caregiver representatives to discuss the importance of attributes and their relevance to the health service in China. During the expert interviews, we will present a list of attributes derived from the literature and qualitative research. Experts will then rank and evaluate these attributes against pre-defined criteria from the perspectives of methodological rigour (DCE experts) and clinical/policy relevance (sexual health experts). The criteria will assess the attribute’s significance to the target population (informed by caregiver input and clinical relevance) and its relevance to potential policy changes (assessed by policymakers and health service researchers). Ultimately, consensus will be reached through group discussion to determine the attributes to be included in the final DCE. The final list of attributes will be determined by their significance to the target population and their relevance to potential policy changes. Example choice scenarios are shown in figures1  2. After determining the attributes and the level of relevance, we will use the software Ngene to generate a D-efficient design to create the choice sets.

**Figure 2:**
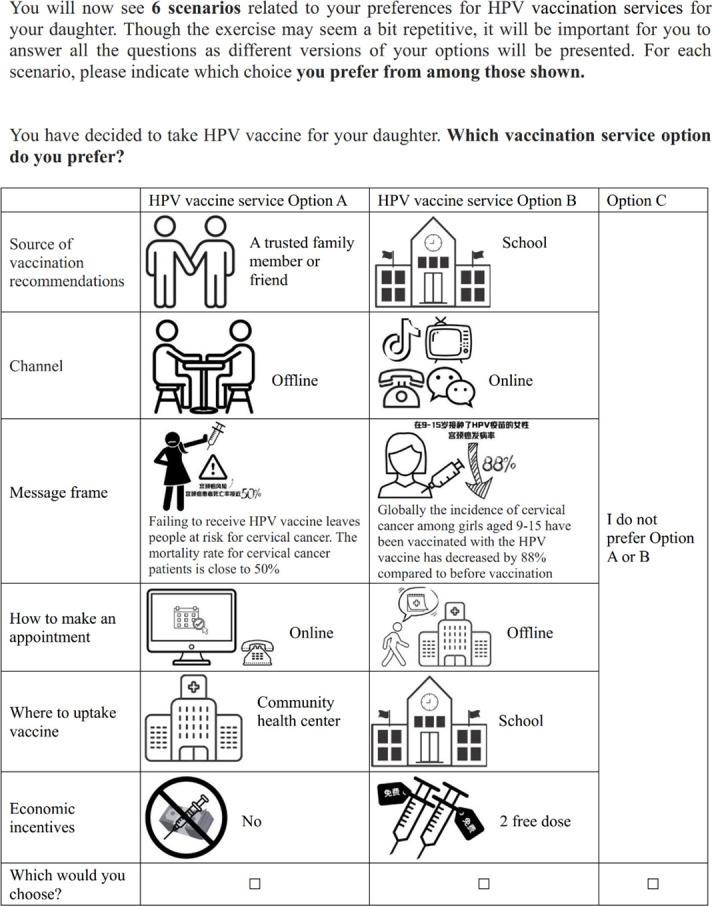
Example of a choice scenario in the discrete choice experiment (B). This scenario presents a hypothetical choice set from the HPV vaccination service discrete choice experiment, illustrating the attributes and levels presented to study participants for evaluation. HPV, human papillomavirus.

**Table 1:** Examples of attributes and levels of the HPV vaccine types in the discrete choice experiment (A)

Attributes	Levels
Level of protection against cervical cancer	70%
	85%
	90%
Level of protection against genital warts	0%
	90%
Risk of severe side effects	1:10 000
	1:100 000
	1:1 000 000
Dose	One
	Two
	Three
Place of manufacture	Local vaccine
	Imported vaccine
Waiting time for vaccination	Access now
	Waiting for 1–12 months
	Waiting for 1–2 years
	Waiting for more than 2 years
Cost (for one dose)	Free
	300RMB
	800RMB
	1300RMB

HPV, human papillomavirus.

**Table 2:** Examples of attributes and levels of HPV vaccination services in the discrete choice experiment (B)

Attributes	Levels
Source of vaccination recommendations	Government
	School
	Physician
	A trusted family member or friend
Channel	Online
	Offline (face to face)
Message frame	Globally, the incidence of cervical cancer among girls aged 9–15 have been vaccinated with the HPV vaccine has decreased by 88% compared with before vaccination.
	Failing to receive the HPV vaccine leaves people at risk of cervical cancer. The mortality rate for cervical cancer patients is close to 50%.
	The WHO and Chinese government recommended that females aged 9 to 14 years should receive HPV vaccine to prevent cervical cancer.
	The WHO and Chinese government recommended that females aged 9 to 45 years should receive HPV vaccine to prevent cervical cancer.
How to make an appointment	Online (website/social media platform/call-in)
	Offline (school/ community healthcare centre)
	Walk-in (no appointment needed)
Where to uptake vaccine	School
	Community healthcare centre
Economic incentives	No incentive
	One free dose
	Two free doses
	One free dose + get a one dose coupon for left-behind girls
	One free dose + get a one dose coupon for someone I know

HPV, human papillomavirus.

##### Blocking

To prevent respondent fatigue, the full set of choice sets is typically divided into smaller subsets (blocks), with each respondent completing only one. Based on the current study design, we anticipate using six blocks, with each block containing 10 choice sets, with four choice sets for the HPV vaccination DCE and six choice sets for the HPV vaccination service DCE.

##### Pilot testing

We intend to recruit a small group of caregivers (approximately 20–30) to pilot-test the face and content validity. Feedback will be collected to further optimise the D-efficient experimental design. Using a think-aloud exercise, which requires participants to verbalise their thoughts in real-time,^17^ we will also check whether respondents understand the tasks and test the cognitive burden. We will seek advice to improve the readability and presentation of the survey. Subsequent changes will be made according to pilot participants’ feedback.

##### Participants and recruitment

Data collection for this study is scheduled to begin in 2024 and conclude in 2025. In addition to the DCEs, we will collect demographic data (age, gender, education, occupation, HPV infection status) and assess respondents’ knowledge, attitudes and intentions regarding HPV vaccination. The survey will use the ‘SoJump’ online survey (https://www.wjx.cn), similar to the Qualtrics survey platform but more commonly used in China, to implement the DCE and collect data. We will conduct this study in Nanjing of Jiangsu Province and Hangzhou of Zhejiang Province (located in eastern China). Differentiating by per capita GDP levels, we will strategically study sites by economic conditions (higher-income, middle-income and lower-income regions). We will then select one district within each economic stratum, totalling three districts. We will collaborate with local community healthcare centres in each district to distribute an invitation link for the online DCE survey through social media platforms (eg, WeChat, an instant messaging platform similar to WhatsApp) and the health information system used by local primary care providers. The health information platform is primarily used by local providers to disseminate health services information, including vaccines. Interested caregivers can voluntarily access the survey by clicking the link and will then be screened for eligibility. Potential participants will be asked to sign an electronic informed consent at the beginning of the survey. Each participant who completes the survey will receive US$3 (or an equivalent value in gifts) as compensation for their time and effort.

### Eligibility criteria

We will recruit persons who meet all of the following criteria:

1. Have at least one daughter aged 9–18 years who has not been vaccinated against HPV.
2. Daughter has no history of vaccination allergy.
3. Understand Chinese and be able to independently complete the survey themselves online.

### Exclusion criteria

1. Daughter has received at least one dose of the HPV vaccine.
2. Daughter has a known history of allergic reaction to vaccines.
3. Daughter is either younger than 9 or older than 18 years old.

### Quality assessment

Screening questions will be set at the beginning of the questionnaire, covering children’s basic information, age, HPV vaccination status and history of allergies to vaccinations. Only respondents who meet all the inclusion criteria can proceed to the formal DCE section. To maximise data utility, we will include data from partially completed questionnaires if their key sections are finished. For instance, if a respondent completed the demographic information and the first DCE task (on HPV vaccine preferences), their data will be included in the analysis of HPV vaccine preferences. Data will be excluded only if critical variables are missing across all essential sections, such as demographics and all DCE tasks. To further ensure the data quality, we will identify ‘speeders’—participants who completed each DCE section significantly faster than the median time—and ‘straight-liners’—who provide identical responses for all DCE sections. We will conduct a sensitivity analysis with and without data from ‘speeders’ and ‘straight-liners’ to assess whether the inclusion of these participants would significantly impact the results.

### Sample size calculation

We used the following parametric-based DCE sample size calculation formula:


n≥500 c/ta

Whereby *n* is the minimal sample size, *t* is the number of choice sets, *a* is the number of alternatives in each choice set and *c* is the highest number of levels of attributes. Based on the above formula, 250 participants will be required for the HPV vaccination DCE survey. The estimation is derived from the inclusion of 4 choice sets per individual, 2 alternatives in each choice set and considering the highest number of levels in the attribute is 4. Approximately 208 participants will be required for the HPV vaccination service DCE survey. The estimation is derived from the inclusion of 6 choice sets per individual, 2 alternatives in each choice set and considering the highest number of levels in the attribute is 5. The final recruitment targets will consider potential drop-out rates. A 20% attrition rate will be used, which aligns with the typical findings from previous online surveys in similar contexts. As a result, the final sample size will be 300.

### Statistical analysis

As the study specifically targets caregivers of girls within a particular age group, it may be difficult to compare participant demographics with local census data. We will systematically record the basic sociodemographic characteristics of individuals who decline participation and compare these profiles with those of enrolled participants and identify systematic differences.

Descriptive analysis is conducted to summarise the key sociodemographic characteristics of the study participants. Multinomial logit (MNL), latent class and mixed logit (MXL) models will be used to assess HPV vaccination preferences and preference heterogeneity. Specifically, the MNL model will be used as the starting point for preliminary analysis. The MXL model with interactions will be used to identify heterogeneity in preferences by subgroup participants (eg, gender, age groups, income levels and perceived behavioural control).^18^ The sign of each attribute level coefficient in the MXL model indicates the direction of its impact on utility (preference), whether positive or negative. Within the same attribute, the magnitude of the coefficient can be used to assess the relative attractiveness of different levels.

To determine the optimal model specification, we will begin with the estimation of a ‘full model’ in which all core attribute parameters are specified as random. The full model will then be compared against simplified versions, where parameters not exhibiting significant heterogeneity are specified as fixed. Model comparison will be based on the Akaike Information Criterion and the Bayesian Information Criterion. In selecting the final model, we will seek a balance between achieving superior model fit and ensuring the theoretical interpretability of the results. Model estimation will be performed using simulated maximum likelihood, with a sufficient number of Halton draws (eg, 1000) to ensure the stability of the estimates. In the MXL model, important demographic variables (eg, gender and education level) will be included as covariates alongside core attribute variables. This will allow us to estimate preferences for attributes while controlling for these demographic factors. Furthermore, subgroup analyses (eg, stratified by education level or income level) will be conducted based on the MXL model to examine whether preferences for attributes vary significantly across different subgroups.

Latent class analysis (LCA), a statistical technique used to identify latent subgroups of respondents based on their response patterns, will be used to examine if there are groups of individuals with similar preferences for HPV vaccination. In this study, LCA will classify respondents into distinct categories with homogeneous preferences, based on their choice patterns. This model will help identify biases associated with different decision-making strategies (eg, consistently selecting the cheapest option or opting out), thereby providing a more nuanced understanding of preference heterogeneity. It will also provide insights into the characteristics of different preference groups, which will inform the design of targeted intervention strategies. All analyses will use effects coding and be performed through NLOGIT (V. 6, Econometric Software Inc, USA), a computer programme designed for analysing choice data.

### Impact

This study will not only evaluate Chinese caregiver preferences for HPV vaccine product characteristics but also offer insights into their preferences regarding vaccine service delivery. The findings from this research will provide crucial insights for policymakers, assisting in the formulation of future vaccine immunisation strategies and guidelines. Additionally, the study contributes to the promotion of vaccines, identification of target vaccine recipients, optimisation of the current distribution of HPV vaccines and guidance for future vaccine research directions.

### Feasibility

Our study team consists of well-experienced experts in various fields, such as sexual health, vaccine delivery, clinical medicine and specialists with extensive experience in DCEs. In addition, our study team has built a strong partnership with the local community healthcare centres through the primary healthcare system in Nanjing. This system, extending across both urban and rural areas in Nanjing, facilitates the provision of immunisation services for local residents in the neighbourhood.

### Ethics and dissemination

Ethical approval was obtained from the Research and Ethics Committee at Nanjing Medical University (approval number (2023) 581). Findings from this study will be disseminated widely to relevant stakeholders via scheduled meetings, webinars, presentations at conferences and in peer-reviewed journal manuscripts.

## Supplementary material

10.1136/bmjopen-2024-089565 online supplemental file 1
