# Supplementary material for: Chinese caregivers’ preferences and predicted uptake of HPV vaccination: a study protocol for two discrete choice experiments
Source: BMJ Open. 2026 Jan 8;16(1):e089565. doi: 10.1136/bmjopen-2024-089565 (PMC13059879; doi:10.1136/bmjopen-2024-089565)
Supplement: online supplemental file 1 [file bmjopen-16-1-s001.docx]

**INTERVIEW OUTLINE**

First of all, thank you for participating in this interview. During the interview, we will ask you to share your knowledge of HPV vaccine vaccination, your decision-making process, and relevant feedback. We would like to record the interview so that we do not miss anything you say. If you do not want the interview to be recorded or wish to stop the recording at any point during the interview, please let us know. If there are no questions, let's begin.

**A. Decision-Making Process for Vaccination**

1. Before participating in the activity related to this interview, what knowledge did you have about the HPV vaccine? Did you have plans to have your child vaccinated with this vaccine? If yes, which valency of the vaccine did you intend to choose?
2. After participating in the program, has this idea changed? What factors have changed your mind?

**B. Financial Subsidies**

1. Will the price of the HPV vaccine be a barrier for you to have your child vaccinated? If it is a barrier, what price range of the HPV vaccine are you willing to accept?
2. What is your opinion on providing certain financial subsidies to promote HPV vaccination among eligible girls?
3. Are there any other forms of financial subsidies that would make you more interested or willing to accept them?

**C. Acquisition and Impact of HPV Vaccine-Related Information**

1. From the HPV vaccine-related promotional materials (such as postcards) provided in this activity, what information did you learn that you did not know before?
2. Which postcard or which piece of content was most helpful to you?
3. How have these pieces of information changed your views on the HPV vaccine and vaccination?
4. What do you think about the role of such promotional information (postcards) in promoting HPV vaccination among eligible girls?

**D. Views on Public Welfare Behaviors Related to Vaccination (Handwritten Postcards and Donations)**

1. If you received a handwritten postcard from another girl, what thoughts or feelings did you have? What do you think is the significance of writing a postcard to the next participant?
2. What is your view on the behavior of "donating money for other eligible girls to receive HPV vaccination"?
3. If you consider participating in donations, what are the main reasons? If you do not consider it, what are your concerns? What factors do you take into account when considering the amount of donation?
4. What do you think about the role of love-passing methods such as handwritten postcards and donations in promoting HPV vaccination among eligible girls?

**E. Feedback and Suggestions on the Implementation Process of Donations, Postcards.**

What do you think about the intervention method of Pay-It-Forward? Are there any aspects that you think can be optimized or reduced?

1. Among financial support, promotional information, and mutual assistance behaviors, which one had the greatest impact on your decision-making? How is this impact specifically reflected?
2. What are your feelings about the overall intervention method of this activity? Which links do you think can be optimized or which contents can be adjusted appropriately?
3. If the activity involves payment methods such as QR code scanning for donations, what is your acceptance of this method? Do you have any questions or concerns about this method? Which method is more acceptable to you (such as anonymous donation, QR code scanning donation, donating to someone you know better, etc.)? Do you think providing different methods will change the amount of your donation?

**SEARCH PARAMETERS**

**Database:** PubMed

**Search Date:** The search was conducted up to Octber, 2023

**Search Terms:**  A combination of keywords and Medical Subject Headings (MeSH) related to three concepts was used:

**HPV/vaccine**: “human papillomavirus”, “HPV”, “cervical cancer”, “HPV vaccines”, “vaccination”

**Preference:** “preference”, “discrete choice experiment”, “DCE”, “conjoint analysis”, “ willingness to pay”, “WTP”

**Population:** “caregiver”, “parents”, “parental preference”

**Study inclusion criteria：**

- Quantitative or qualitative research, systematic reviews, meta-analyses
- Study subjects: parents, caregivers
- Content focus: Explicitly reporting data concerning HPV vaccination preferences, willingness or choice tendencies

**Key finding:**

**Figure 1** List of Identified Attributes from Literature Review

| Attributes | Levels | Reference |
| --- | --- | --- |
| Protection against cervical cancer | 50% / 70% / 90% / 100% | Brown et al. 2010 |
|  | 90% / 95% / 98% / 100% | Oteng et al. 2010 |
|  | 50% / 70% / 90% | Poulos et al. 2011 |
|  | 50% / 70% / 80% / 905 | Wong et al. 2018 |
|  | 50% / 70% / 90% | Zhu et al. 2020 |
| Protection against genital warts | No protection / 90% | Brown et al. 2010 |
|  | No protection / 90% / 95% / 98% | Oteng et al . 2010 |
| Duration of protection | 2yrs / 5yrs / 10yrs / lifetime | Brown et al. 2010 |
|  | 2yrs / 10yrs / lifetime | Poulos et al. 2011 |
|  | 2yrs / 5yrs / lifetime | Wong et al. 2018 |
|  | 6yrs / 25yrs / lifetime | Zhu et al. 2020 |
| Side effect | 2:100 / 6:100 /10:100 / 14:100 | Oteng et al. 2010 |
|  | 2:100 / 6:100 /10:100 / 14:100 | Wong et al. 2018 |
|  | 1/50 / 1/30 / 1/10 | Zhu et al. 2020 |
| Out-of-pocket cost | 0 / $100 / $300 / $700 | Brown et al. 2010 |
|  | 0 / $200 / $400 / $600 | Oteng et al. 2010 |
|  | $6 / $29 / $118 / $353 | Poulos et al. 2011 |
|  | 0 / 1000HKD / 2000HKD / 2000 HKD / 3000HKD | Wong et al. 2018 |
|  | 900CNY / 1800CNY / 2700CNY | Zhu et al. 2020 |
| Vaccination location | Vaccination center / school | Zhu et al. 2020 |
