# Supplementary material for: Chinese caregivers’ preferences and predicted uptake of HPV vaccination: a study protocol for two discrete choice experiments
Source: BMJ Open. 2026 Jan 8;16(1):e089565. doi: 10.1136/bmjopen-2024-089565 (PMC13059879; doi:10.1136/bmjopen-2024-089565)
Supplement: Supplementary file 2 [file bmjopen-2024-089565.reviewer_comments.pdf]

PEER REVIEW HISTORY

BMJ Open publishes all reviews undertaken for accepted manuscripts. Reviewers are asked to complete a checklist review form and are provided with free text boxes to elaborate on their assessment. These free text comments are reproduced below.

ARTICLE DETAILS

Title (Provisional)

Chinese Caregivers' Preferences and Predicted Uptake of HPV Vaccination: a Study Protocol for two Discrete Choice Experiments

Authors

Fang, Han; Mou, Jialing; Chow, Eric PF; Ong, Jason J.; Wu, Dan; Zhang, Ye

---

VERSION 1 - REVIEW

**Reviewer**	**1**
**Name**	**Bonner, Kimberly E.**
**Affiliation**	**University of Minnesota System**
**Date**	**03-Oct-2024**
**COI**	**None**

BMJ Open

Preferences for Chinese Caregivers for their daughters' HPV vaccines and services: a study protocol for two discrete choice experiments

The authors pose two timely and important questions regarding preferred HPV vaccine profile and preferred service delivery modality among caregivers of girls aged 9-18 years in China. While HPV vaccination has been available in China since 2016, the vaccine is not on the routine immunization program in China, and vaccination rates in the 9-18 years age group of girls are less than 1%. The discrete choice experiment modality permits the assessment of stated preferences for vaccine profiles as well as for service delivery modalities among caregivers of vaccine-eligible girls in an effort to inform decision-making on strategies to increase vaccination uptake. This study design is appropriate and the protocol is clearly written.

Please note the following feedback

Major:

- Consider the systematic differences between caregivers of girls who received HPV vaccination compared to those who had not received HPV vaccination. If only 1% of the eligible population has received the HPV vaccine, consider shifting the qualitative assessment focus group discussions to caregivers of girls who have not received the HPV vaccine. (Page 6, lines 18-21)

- Given the opt-in recruitment modalities, consider including a means to identify potential selection bias among respondents in the analysis, particularly if you plan to generalize the findings to the entire geographic area.

- If the authors plan to assess whether a particular attribute (level) affects the choices that respondents make, consider using the sample calculation provided by de Bekker-Grob, E.W., Donkers, B., Jonker, M.F. et al., which includes r code for sample size calculation: Sample Size Requirements for Discrete-Choice Experiments in Healthcare: a Practical Guide. Patient 8, 373–384 (2015). https://doi.org/10.1007/s40271-015-0118-z

- If the authors plan to make any comparisons between districts or socioeconomic strata, consider that the sample size will need to updated to reflect this multi-level analysis.

- Please explain how the authors plan to run the mixed logit model (e.g. note fixed and random effects) and please note how these findings are meant to be interpreted. Do the authors plan to identify the attributes that drive decision-making? Or will the LCA model create the groups for comparison in the subsequent mixed logit models?

Minor:

- Please include a citation for “previous studies have examined parents’ preference for HPV vaccination” (page 5, line 23)

- Please include a citation for random utility theory (page 5, line 43-44)

- Please include the survey platform modality that will be used to collected the data (e.g. Qualtrics?)

- Please note if and how eligibility will be verified in these self-administered surveys

- Five levels can be difficult to efficiently block. Consider using 4 or 6.

- Please clarify if respondents need to complete the survey for their data to be included or if partially completed surveys will be included in the analysis

- Please consider updating exclusion criteria (page 9 line 2) to indicate if “vaccinated” means at least one dose

- Consider waiting time to receive a vaccine at the point of delivery in the assessment of service delivery attributes. Consider updating the risk of severe side effects in a manner that would be more intuitive to the respondent (e.g. one person in your district would likely receive a severe side effect).

- Rumors and misinformation have been a persistent deterrent of HPV vaccination. Consider if these channels or messages need to be weighed in these DCEs.

**Reviewer**	**2**
**Name**	**Lingas, Elena**
**Affiliation**	**Not Applicable**

**Date**	**16-Dec-2024**
**COI**	**None**

Review of bmjopen-2024-089565: Preferences of Chinese caregivers for their daughters' HPV vaccines and services: a study protocol for two discrete choice experiments

The authors have submitted a paper to share their protocol; as written, the manuscript does not provide enough information for others to be able to understand how this study will be conducted, nor to replicate said study. The following suggestions are made to strengthen the protocol.

Introduction

The background would benefit from greater detail about the health context in Nanjing, where it is revealed much later in the paper that the study will take place. What is the incidence rate of HPV-caused cervical cancer? What is the health care delivery and policy context—how has the roll-out of vaccines gone in this particular area? The authors indicate that their study is needed to improve service delivery and resource allocation, but do not provide enough detail to understand the service delivery and resource allocation context. Consequently, there is not a clear rationale/need for this study.

Methods

When will this study be taking place? There is no information about dates/timelines. It is also unclear how "consumers" and the public will be involved in the study—again, information about the local health care delivery context and communication channels are needed.

Qualitative

The declared focus of the protocol is to conduct discrete choice experiments (DCE), and yet the first part of the methods includes semi-structured interviews, which are also described as focus group discussions. Focus groups and semi-structured interviews are two different qualitative methods, and cannot be considered interchangeable. Also, the authors propose to potentially conduct an online focus group depending on participant preference; it is unclear how a true focus group would work in an online environment. Where is the draft interview and/or focus group guide? Also, the need for this data collection step, however it is conducted, is not clear—what does this step have to do with the DCE? As for the analysis of this stage, it would be useful to know who among the authors (or if it will be others not listed) will be coding the transcripts, and what the "potential themes" are that they plan to explore.

Finally, with regard to data management, the authors state that they will undergo "training and ethics certification," and yet this protocol states in the abstract that it has been approved by the "Research and Ethics Committee at Nanjing Medical University." Does this mean human subjects training is not required for investigators prior to institutional research approval? This issue requires further clarification.

DCE

A "comprehensive review" of two types of literature is mentioned; please include the search parameters and findings of these reviews as they undergird this part of the protocol. It seems that the authors intend to conduct further interviews with "DCE design experts and sexual health experts;" this data collection step needs to be further explained. It is unclear how the list of DCE attributes will be determined other than by "their significance to the target population and their relevance to potential policy changes"—how these ideas will be operationalized requires further explanation.

The authors go on to state that they will pilot test the DCE—is the recruitment plan listed for the pilot testing or for the actual DCE experiment? Please explain and provide a citation for a "think-aloud exercise." The DCE process needs further explanation for those unfamiliar with the terminology of "blocks," "choice sets," and "levels of attributes." Also, why do the authors think they need to stratify recruitment by regional income level? What has the literature shown about this impact on HPV vaccination in China?

Regarding sample size calculations, will the sample be increased to account for attrition? What additional demographic data will be collected from the participants? Please explain "perceived behavioral control" as this is one of the types of data that will be collected—what does this mean/what is its relevance for this study? Please spell out/explain what an "LCA model" is and its relevance to this study.

VERSION 1 - AUTHOR RESPONSE

Reviewer 1 comments

We are sincerely grateful to Reviewer 1 for acknowledging the significance and design of our research, as well as for their insightful and constructive comments. Below is our point-by-point response to Reviewer 1.

Major:

1. **Consider the systematic differences between caregivers of girls who received HPV vaccination compared to those who had not received HPV vaccination. If only 1% of the eligible population has received the HPV vaccine, consider shifting the qualitative assessment focus group discussions to caregivers of girls who have not received the HPV vaccine. (Page 6, lines 18-21)**

Response: We agree that understanding the perspectives of caregivers of unvaccinated girls is crucial for addressing vaccine hesitancy. Numerous studies have focused on identifying barriers and influencing factors among unvaccinated girls and caregivers in China. But there is a lack of in-depth evidence on the facilitators and drivers from the perspective of those who have successfully completed the vaccination process. To fix the gap of previous literature, the primary aim of our qualitative study is to identify these facilitators and positive drivers behind successful HPV vaccination. By specifically recruiting caregivers who have already vaccinated their daughters, we can gain deeper insights into the decision-making process. This includes understanding the key influencers (e.g., healthcare providers, school programs) and the strategies used to overcome barriers, all from the perspective of those who have successfully adopted the vaccine.

2. **Given the opt-in recruitment modalities, consider including a means to identify potential selection bias among respondents in the analysis, particularly if you plan to generalize the findings to the entire geographic area.**

Response: Thanks for raising this important issue. We have provided additional clarification in the "Statistical Analysis" section (page10). As the study specifically targets caregivers of girls within a particular age group, it may be difficult to compare participant demographics with census data from the local setting. We will record the characteristics of individuals who decline to participate in the study and compare their sociodemographic profiles with those who choose to participate. In the final report, we will carefully discuss the external validity of the findings and acknowledge the limitations of our sample to avoid overgeneralization.

"*As the study specifically targets caregivers of girls within a particular age group, it may be difficult to compare participant demographics with local census data. We will systematically record the basic sociodemographic characteristics of individuals who decline participation and compare these profiles with those of enrolled participants and identify systematic differences.*"

If the authors plan to assess whether a particular attribute (level) affects the choices that respondents make, consider using the sample calculation provided by de Bekker-Grob, E.W., Donkers, B., Jonker, M.F. et al., which includes r code for sample size calculation: Sample Size Requirements for Discrete-Choice Experiments in Healthcare: a Practical Guide. Patient 8, 373–384 (2015). https://doi.org/10.1007/s40271-015-0118-z

3. **If the authors plan to make any comparisons between districts or socioeconomic strata, consider that the sample size will need to updated to reflect this multi-level analysis.**

Response: We sincerely thank the reviewer for suggesting the sample size calculation method from de Bekker-Grob et al. (2015). We carefully followed the formula provided, however, the calculation for one of our attribute levels indicated a minimum requirement of approximately 1,300 respondents. Given the practical difficulties in recruiting such a large sample within our study context, we ultimately adopted the widely used rule-of-thumb approach from Johnson and Orme (2003). This method is commonly applied in DCE studies, as shown in studies on health interventions in Germany and on HPV vaccination preferences in Hong Kong [1, 2]. We are confident that the sample size based on this approach will be adequate to provide reliable and stable parameter estimates for our model.

[1]. Phillips EA, Himmler SF, Schreyögg J. Preferences for e-Mental Health Interventions in Germany: A Discrete Choice Experiment. Value Health. 2021 Mar;24(3):421-430. doi: 10.1016/j.jval.2020.09.018.

[2]. Wong CKH, Man KKC, Ip P, Kwan M, McGhee SM. Mothers' Preferences and Willingness to Pay for Human Papillomavirus Vaccination for Their Daughters: A Discrete Choice Experiment in Hong Kong. Value Health. 2018 May;21(5):622-629. doi:

10.1016/j.jval.2017.10.012.

4. **Please explain how the authors plan to run the mixed logit model (e.g. note fixed and random effects) and please note how these findings are meant to be interpreted. Do the authors plan to identify the attributes that drive decision- making? Or will the LCA model create the groups for comparison in the subsequent mixed logit models?**

Response: We have provided detailed schedule for running the mixed logit model in the "Statistical Analysis" section (page 10). The expanded content consists of model specification and result interpretation. We will employ the LCA model to create the groups for comparison in the subsequent mixed logit models. Specific analytical steps have also been incorporated into the "Statistical analysis" section.

"*The sign of each attribute level coefficient in the MXL model indicates the direction of its impact on utility (preference), whether positive or negative. Within the same attribute, the magnitude of the coefficient can be used to assess the relative attractiveness of different levels.*"

"*To determine the optimal model specification, we will begin with the estimation of a "full model" in which all core attribute parameters are specified as random. The full model will then be compared against simplified versions, where parameters not exhibiting significant heterogeneity are specified as fixed. Model comparison will be based on the Akaike Information Criterion(AIC) and the Bayesian Information Criterion(BIC). In selecting the final model, we will seek a balance between achieving superior model fit and ensuring the theoretical interpretability of the results. Model estimation will be performed using simulated maximum likelihood, with a sufficient number of Halton draws (e.g., 1000) to ensure the stability of the estimates. In the MXL model, important demographic variables (e.g., gender, education level) will be included as covariates alongside core attribute variables. This will allow us to estimate preferences for attributes while controlling for these demographic factors. Furthermore, subgroup analyses (e.g., stratified by education level or income level) will be conducted based on the MXL model to examine whether preferences for attributes vary significantly across different subgroups.* "

"*Latent Class Analysis (LCA) , a statistical technique used to identify latent subgroups of respondents based on their response patterns,* will be used to examine if there are groups of individuals with similar preferences for HPV vaccination. *In this study, LCA will classify respondents into distinct categories with homogeneous preferences, based on their choice patterns. This model will help identify biases associated with different decision-making*

strategies (e.g., consistently selecting the cheapest option or opting out), thereby providing a more nuanced understanding of preference heterogeneity. It will also provide insights into the characteristics of different preference groups, which will inform the design of targeted intervention strategies."

Minor:

1. **Please include a citation for "previous studies have examined parents' preference for HPV vaccination" (page 5, line 23)**

Response: We have added a citation for this statement.

[6] Zhu S, Chang J, Hayat K, et al. Parental preferences for HPV vaccination in junior middle school girls in China: A discrete choice experiment. Vaccine. 2020;38:8310–7. doi: 10.1016/j.vaccine.2020.11.020

[15] Lack A, Hiligsmann M, Bloem P, *et al.* Parent, provider and vaccinee preferences for HPV vaccination: A systematic review of discrete choice experiments - ScienceDirect. *Vaccine*. 2020;38:7226–38. doi: 10.1016/j.vaccine.2020.08.078

2. **Please include a citation for random utility theory (page 5, line 43-44)**

Response: We have added a citation for this statement.

[16] Lancsar E, Louviere J. Conducting discrete choice experiments to inform healthcare decision making: a user's guide. *PharmacoEconomics*. 2008;26:661–77. doi: 10.2165/00019053-200826080-00004

3. **Please include the survey platform modality that will be used to collected the data (e.g. Qualtrics?)**

Response: We have added in the "Participants and Recruitment" section (page 8) that this study will use the "SoJump" online survey platform, similar to the Qualtrics survey platform but more commonly used in China, to implement the DCE and collect data.

"*The survey will use the "SoJump" online survey (https://www.wjx.cn), similar to the Qualtrics survey platform but more commonly used in China, to implement the DCE and collect data.*"

4. **Please note if and how eligibility will be verified in these self-administered surveys**

   Response: In the "Quality assessment" section (page 9), we have added the following description: "*Screening questions will be set at the beginning of the questionnaire, covering children's basic information, age, HPV vaccination status, and history of allergies to vaccinations. Only respondents who meet all the inclusion criteria can proceed to the formal DCE section.*"

5. **Five levels can be difficult to efficiently block. Consider using 4 or 6.**

Response: Thank you for your professional suggestions. We are currently presenting a design proposal. The final DCE design will be determined based on qualitative interviews and discussions with key stakeholders.

6. **Please clarify if respondents need to complete the survey for their data to be included or if partially completed surveys will be included in the analysis**

Response: We have clearly specified in the "Quality Assessment" section (page9).

"*To maximize data utility, we will include data from partially completed questionnaires if their key sections are finished. For instance, if a respondent completed the demographic information and the first DCE task (on HPV vaccine preferences), their data will be included in the analysis of HPV vaccine preferences. Data will be excluded only if critical variables are missing across all essential sections, such as demographics and all DCE tasks.*"

7. **Please consider updating exclusion criteria (page 9 line 2) to indicate if "vaccinated" means at least one dose**

Response: We have updated the exclusion criteria and clearly defined "vaccinated" as having received at least one dose of the HPV vaccine (page 9).

"Daughter has been *received at least one dose of the HPV vaccine.*"

8. **Consider waiting time to receive a vaccine at the point of delivery in the assessment of service delivery attributes. Consider updating the risk of severe side effects in a manner that would be more intuitive to the respondent (e.g. one person in your district would likely receive a severe side effect).**

Response: We thank the reviewer for these thoughtful suggestions. Our prior qualitative research and a review of the standard service flow in Chinese vaccination clinics suggested that the waiting time at vaccination clinics is minimal and not a salient factor influencing caregivers' decision-making. The process has been reported to be well organized, and once an appointment is secured, the on-site administration will be quite efficient. Therefore, we decided to keep the DCE focused on the most contextually relevant drivers of preference, and prioritized other service delivery attributes, such as the location and the availability of appointments, which are more likely to be identified as primary concerns.

In order to enhance the intuitiveness of presenting severe side effect risks. While a district-based framework might offer intuitive appeal, we chose to use a consistent numerical probability in our discrete choice experiment to avoid potential ambiguity arising from population size differences across districts. Such differences could lead to inconsistent risk perceptions among respondents, thereby introducing bias into the choice data and complicating the interpretation of preference estimates. Moreover, this numerical format was tested in our pilot study, where respondents confirmed it was clear and understandable. Based on this feedback, we believe our current approach provides a consistent and interpretable measure of risk preferences.

9. **Rumors and misinformation have been a persistent deterrent of HPV vaccination. Consider if these channels or messages need to be weighed in these DCEs.**

Response: We agree that this is a critical factor influencing HPV vaccination decisions. Our questionnaire includes questions about caregivers' vaccine hesitancy, refusal behavior, and we will include misinformation as one of the key reasons for hesitancy. This allows us to capture whether rumors or misinformation lead to their negative attitudes toward HPV vaccination.

Reviewer 2 comments

We extend our sincere gratitude to Reviewer 2 for the meticulous and insightful comments, which have greatly assisted us in refining our research design, rendering it clearer and more rigorous.

1. **Introduction: The background would benefit from greater detail about the health context in Nanjing, where it is revealed much later in the paper that the study will take place. What is the incidence rate of HPV-caused cervical cancer? What is the health care delivery and policy context—how has the roll-out of vaccines gone in this particular area? The authors indicate that their study is needed to improve service delivery and resource allocation, but do not provide enough detail to understand the service delivery and resource allocation context. Consequently, there is not a clear rationale/need for this study.**

Response: We fully agree with your view that detailed local context is crucial for clarifying the necessity of this study. Therefore, we have added a new paragraph in the Background section (Page 4 of the revised manuscript), with specific supplements as follows:

"*Cervical cancer ranked second among female cancers incidence in Jiangsu Province and sixth in Zhejiang Province. To reduce this disease burden and advance HPV vaccination efforts, both Jiangsu and Zhejiang began offering free domestic bivalent HPV vaccinations to eligible 14-year-old girls in 2022, which substantially increase HPV vaccination rate among this age group. However, the current vaccination efforts face two prominent challenges. First, the vaccination rate among first-grade junior high school girls is gradually reaching a plateau, creating a pressing need to surpass the current coverage level. Second, the existing policy exclusively targets 14-year-old adolescent females, leaving a gap in mobilization mechanisms for girls aged 15 years, whose participation also requires focused attention. These practical challenges highlight the urgency and necessity of conducting in-depth research in pioneering cities like Nanjing and Hangzhou to optimize service delivery models and resource allocation efficiency.*"

2. **When will this study be taking place? There is no information about dates/timelines. It is also unclear how "consumers" and the public will be involved in the study—again, information about the local health care delivery context and communication channels are needed.**

Response: We have added the research timeline at the beginning of the "Participants and Recruitment" section (page 8): "*Data collection for this study is scheduled to begin in 2024 and conclude in 2025.*"

We have revised the PPI statement as instructed (page 5).

"*Consumers and key stakeholders will be involved in finalizing the attributes for the DCE. Their input will provided valuable feedback on the relevance, clarity, and classification of potential attributes, ensuring that these attributes accurately reflect their needs and perceptions. They will also contribute to optimizing the final presentation of the questionnaire, making it clearer and easier to understand.*"

3. **Qualitative:**

- **Focus groups and semi-structured interviews are two different qualitative methods, and cannot be considered interchangeable.**

how a true focus group would work in an online environment. Where is the draft interview and/ or focus group guide?

Response: Thank you for flagging this error, we have amended it accordingly(page 5). We have also clarified how a group discussion would work in an online environment.

"Qualitative data will be gathered through a total of four *semi-structured group* discussions, with 6-8 interviewees for each group discussion(*The interview outline can be found in the supplementary File1*). Study participants will be purposively sampled among caregivers of eligible girls (9-18 years) who have either been vaccinated or plan to receive the HPV vaccine. Social media platforms and health information systems of community health centers will be used to recruit participants. Individuals expressing interest will be contacted by the research team skilled in community engagement and qualitative research. Interviews will be conducted through either online or in-person meetings, depending on participant preference. *The online group will use a professional video conferencing software (Tencent Meeting) to conduct the session. The host will control the flow, and the chatroom and raise hand features will simulate in-person interaction.*"

- **what does this step have to do with the DCE?**

Response: We conducted semi-structured interviews primarily to identify the attributes and levels associated with HPV vaccination decision-making. For example, interview findings showed that the incentive of providing a single-dose coupon to left-behind girls could also influence parents' vaccination decisions, so we incorporated this as a level under the "incentive" attribute. Group interviews further helped us understand parents' considerations, information gaps, and trade-off processes when making HPV vaccination decisions, ensuring the real-world relevance of the research results. Additionally, they assisted in optimizing the expression and presentation of the DCE questionnaire, enabling the translation of some jargons into user-friendly language.

- **As for the analysis of this stage, it would be useful to know who among the authors (or if it will be others not listed) will be coding the transcripts, and what the "potential themes" are that they plan to explore.**

Response: Thank you for raising this point. The qualitative interviews are pending, and the coding will be carried out by trained qualitative researchers among our author team. The process will involve independent coding followed by discussions to reach consensus, ensuring the reliability of the analysis.

We have elaborated on the "Qualitative Data Analysis" section (page 6) by adding the key potential themes for exploration.

"Potential themes will be explored based on previous literature, and emerging new themes from raw data will also be generated. *The potential themes include Vaccine Cognition (e.g., respondents' prior understanding of HPV vaccines), Price and Subsidy(e.g., whether vaccine prices are a barrier to vaccination, acceptable price ranges, attitudes toward economic subsidies, and preferred subsidy forms), and community engagement and support for other people.* Two coders will then review all transcripts and apply the coding framework to analyze the data independently, utilizing NVivo to organize the data."

- **Finally, with regard to data management, the authors state that they will undergo "training and ethics certification," and yet this protocol states in the abstract that it has been approved by the "Research and Ethics Committee at Nanjing Medical University." Does this mean human subjects training is not required for investigators prior to institutional research approval? This issue requires further clarification.**

Response: Sorry about the confusing language. All group members have received ethics training already and we revised the language to avoid confusion. The PIs of the study completed the Human Research Social and Behavioral Research course via the Collaborative Institutional Training Initiative (CITI Program) and all group members must complete the ethics training module as a mandatory course offered by the university. We further confirm that all investigators fully understand and commit to upholding the highest ethical principles throughout this research.

4. DCE:

- **A "comprehensive review" of two types of literature is mentioned; please include the search parameters and findings of these reviews as they undergird this part of the protocol.**

Response: We thank the reviewer for this suggestion. We have now created a comprehensive supplementary file (supplementary File1) that details the search parameters and summarizes the key findings . This file has been submitted alongside the revised manuscript.

- **It seems that the authors intend to conduct further interviews with "DCE design experts and sexual health experts;" this data collection step needs to be further**

explained. It is unclear how the list of DCE attributes will be determined other than by "their significance to the target population and their relevance to potential policy changes"—how these ideas will be operationalized requires further explanation

Response: We added the further explain in "Experimental design" section(page 7). "*During the expert interviews, we will present a list of attributes derived from the literature and qualitative research. Experts will then rank and evaluate these attributes against pre-defined criteria from the perspectives of methodological rigor (DCE experts) and clinical/policy relevance (sexual health experts). The criteria will assess the attribute's significance to the target population (informed by caregiver input and clinical relevance) and its relevance to potential policy changes (assessed by policymakers and health service researchers). Ultimately, consensus will be reached through group discussion to determine the attributes to be included in the final DCE.* The final list of attributes and will be determined by their significance to the target population and their relevance to potential policy changes."

- **The authors go on to state that they will pilot test the DCE—is the recruitment plan listed for the pilot testing or for the actual DCE experiment?**

Response: The recruitment plan outlined in the "pilot testing" section was specific to that phase. The detailed recruitment strategy for the formal Discrete Choice Experiment (DCE) is subject to revisions based on our pilot testing outputs described separately in the "Participants and Recruitment" section of the manuscript.

- **Explain and provide a citation for a "Think-aloud exercise"**

Response: We have added an explanation and reference citation for the think-aloud exercise in the "pilot testing" section(page8).

"Using a think-aloud exercise, *which requires participants to verbalize their thoughts in real-time,* we will also check whether respondents understand the tasks and test the cognitive burden."

- **The DCE process needs further explanation for those unfamiliar with the terminology of "blocks," "choice sets," and "levels of attributes."**

Response: We have supplemented explanations for these terms where they first appear in the text (in the "Overview of approach and methods" section on page 5 and the blocking section on page 8).

"In a DCE, each participant faces a series of choice *sets(In a choice task, the complete set of options presented to respondents for trade-off comparisons,* usually with two alternatives). Each choice set consists of several attributes*(i.e., The characteristics of an option, such as "cost")* that ask respondents to trade-off between preferred and less preferred attribute levels*(i.e., the specific values that each attribute takes, such as "CNY300", "CNY800")* present in each choice task."

"*To prevent respondent fatigue, the full set of choice sets is typically divided into smaller subsets (blocks), with each respondent completing only one.* Based on the current study design, we anticipate using 6 blocks, with each block containing 10 choice sets, with 4 choice sets for the HPV vaccination DCE and 6 choice sets for the HPV vaccination service DCE."

- **Why do the authors think they need to stratify recruitment by regional income level? What has the literature shown about this impact on HPV vaccination in China**

Response: We ideally planned to conduct stratified recruitment by regional income level, as existing studies have shown that household income is a key factor influencing the willingness to pay for HPV vaccines and vaccination decisions. This sampling method would ensure that our sample better represents the population from different socioeconomic backgrounds, thereby enhancing the generalizability of the results. The final recruitment strategy will be finalized based on the outcomes of our pilot testing.

- **Regarding sample size calculations, will the sample be increased to account for attrition?**

Response: Yes, using an empirical approach, we calculated the minimum sample size to be 250 participants. Considering a 20% attrition rate, the final sample size will be 300 participants. We have updated the sample size in the "Sample Size Calculation" section (page 10) accordingly.

"The final recruitment targets will consider potential drop-out rates. A 20% attrition rate will be used, which aligns with the typical findings from previous online surveys in similar contexts. *As a result, the final sample size will be 300.*"

- **What additional demographic data will be collected from the participants?**

Response: In addition to the DCEs, we will collect demographic data (age, gender, education, occupation, HPV infection status) and assess respondents' knowledge, attitudes, and intentions regarding HPV vaccination. We have added this information in the "participants and recruitment" section.(page 8)

"Data collection for this study is scheduled to begin in 2024 and conclude in 2025. *In addition to the DCEs, we will collect demographic data (age, gender, education, occupation, HPV infection status) and assess respondents' knowledge, attitudes, and intentions regarding HPV vaccination.*"

- **Please explain "perceived behavioral control" as this is one of the types of data that will be collected—what does this mean/what is its relevance for this study?**

  Response: Perceived Behavioral Control (PBC) refers to a caregiver's perception of how easy or difficult it is to get their daughter vaccinated against HPV, encompassing factors like cost, convenience, and access. In this study, PBC is relevant because we aim to explore whether parents' perceptions of the ease or difficulty of getting the vaccine influence their decision-making.

- **Please spell out/explain what an "LCA model" is and its relevance to this study**

  Response: Latent Class Analysis (LCA) is a statistical model used to identify latent subgroups of respondents based on their response patterns. In this study, we will employ LCA to classify respondents into several categories with homogeneous preferences, based on their choice patterns. This approach will facilitate a deeper understanding of the characteristics of different preference groups, providing a foundation for the development of targeted intervention strategies. We have included the aforementioned information in the "Statistical Analysis" section.

"*Latent Class Analysis (LCA) , a statistical technique used to identify latent subgroups of respondents based on their response patterns,* will be used to examine if there are groups of individuals with similar preferences for HPV vaccination. *In this study, LCA will classify respondents into distinct categories with homogeneous preferences, based on their choice patterns. This model will help identify biases associated with different decision-making strategies (e.g., consistently selecting the cheapest option or opting out), thereby providing a more nuanced understanding of preference heterogeneity. It will also provide insights into the characteristics of different preference groups, which will inform the design of targeted intervention strategies.* "

Once again, we extend our sincere gratitude for your time and the gracious guidance provided throughout the review process. We are hopeful that our revisions have appropriately addressed all the points raised, and we eagerly await your further feedback.
